# Los errores en las pruebas de cabecera pueden resultar en falsos niveles de potasio

**DOI:** 10.1515/almed-2022-0015

**Published:** 2022-03-01

**Authors:** Antonio Buño Soto, Paloma Oliver Sáez

**Affiliations:** Departamento de análisis clínicos, Hospital Universitario La Paz, Madrid, España

**Keywords:** pruebas de cabecera (POCT), hemólisis, gasometría, pseudohiperpotasemia

## Abstract

Las pruebas de cabecera (POCT, por sus siglas en inglés) permiten disponer de los resultados en un corto espacio de tiempo, facilitando la toma rápida de decisiones médicas. Sus resultados deben ser fiables, y su calidad no debe verse comprometida durante el proceso. Las gasometrías son una de las pruebas POCT más utilizadas en los servicios de urgencias y cuidados intensivos. Se suele utilizar sangre entera como muestra, debiendo tener en cuenta el riesgo de hemólisis. Sin embargo, los analizadores empleados para las gasometrías POCT no detectan la presencia de hemólisis en la muestra y, debido a las características de la misma, tampoco se puede identificar la hemólisis a simple vista. La hemólisis puede alterar el resultado de parámetros como el potasio, mostrando resultados anormalmente elevados o concentraciones normales, enmascarando niveles que, en realidad, son bajos (hipopotasemia). La hiperpotasemia está asociada a un mayor riesgo de sufrir arritmia potencialmente fatal, y requiere de una actuación médica urgente. La hemólisis es la causa más frecuente de pseudohiperpotasemia (hiperpotasemia espuria) o pseudonormopotasemia, lo que puede llevar a un diagnóstico erróneo y a la consiguiente toma de decisiones clínicas inadecuadas. En el presente artículo, realizamos una revisión completa de los posibles factores que pueden hacer que una prueba POCT informe un resultado falso de concentraciones elevadas de potasio en sangre. Los programas de POCT adecuadamente supervisados y organizados por el laboratorio clínico pueden contribuir a prevenir errores y reducir su impacto en el manejo del paciente.

## Introducción

Price y Hick definen las pruebas de cabecera (POCT) como aquellos análisis de sangre y otro fluidos corporales, o estudios de suspensiones y extensiones de células y cristales, que no se realizan en el laboratorio clínico central o en un laboratorio satélite, sino que se llevan a cabo cerca del paciente, con el propósito de disponer de los resultados de manera inmediata o en un plazo muy corto de tiempo, lo que permitirá realizar los cambios oportunos en el manejo del paciente, si procede [1].

El motivo principal por el que se emplean las POCT es que estas permiten disponer de los resultados en un periodo limitado de tiempo, ya que las muestras no se envían a un laboratorio central, lo que contribuye a una rápida toma de decisiones clínicas. Es esencial que los resultados de las POCT sean fiables para optimizar el manejo de los pacientes. Aunque no es necesario que los profesionales sanitarios que realizan estas pruebas dispongan de titulación en análisis clínicos, estos deben haber recibido formación y certificación por parte de un profesional de laboratorio, que deberá supervisar esta parte crucial en el proceso de realización de las POCT. A pesar de sus posibles beneficios, como un tiempo de respuesta rápido, o un menor volumen de errores preanalíticos debido a la simplificación de los procesos, su impacto no ha sido suficientemente estudiado. Cada vez existe una mayor disponibilidad de dispositivos POCT, en términos de variedad de pruebas, número de proveedores y disponibilidad de métodos y dispositivos y aún se espera que su uso continúe incrementándose [2].

La Organización Mundial de la Salud define error médico como un evento adverso o incidente que sería evitable con los conocimientos médicos actuales. Un evento adverso asociado al uso de un dispositivo es un incidente en el que la utilización de un equipo médico podría haber perjudicado la salud del paciente. Un evento adverso evitable es un evento adverso que podría haberse evitado si se hubieran seguido los estándares de asistencia habituales en ese momento [3]. Los errores médicos en los análisis clínicos suelen deberse a fallos en las acciones analíticas previstas, entre las que se incluyen las POCT [4]. En un estudio sobre errores en laboratorios de urgencias publicado por Plebani y cols. en 1997, de 189 errores detectados por profesionales sanitarios, tres cuartas partes de los mismos (140 [70%]) no perjudicaron la salud de los pacientes, aunque una quinta parte (37 [19.6%]) derivaron en pruebas innecesarias, lo que implicó un incremento injustificado de costes. Por otro lado, 1 de cada 16 (12 [6.3%]) derivaron en una actuación médica inadecuada o una modificación incorrecta de tratamiento [5].

Los errores en las POCT se pueden clasificar según la fase del proceso en la que se producen, a saber: preanalíticos, analíticos y postanalíticos. La mayoría de ellos se producen en la fase preanalítica, como es el caso de los errores en los laboratorios de urgencias, donde, según algunos autores, el 68.2% de los errores son preanalíticos, el 13.3% son analíticos y el 18.5% son postanalíticos [5]. En la fase preanalítica, existen varios pasos en los que se pueden producir errores, como en la solicitud de la prueba, la identificación del paciente o de la muestra, la obtención de la muestra, en la que se incluya un tipo de muestra inapropiado, o volumen incorrecto de muestra, o una aplicación incorrecta sobre la superficie la tira o cámara de reacción del dispositivo de POCT; por último, se pueden producir errores en la evaluación de la muestra o de sus características. Kost distingue entre dos tipos de atributos que pueden influir en los análisis de sangre entera: aquellos relacionados con el paciente, como la anemia o la leucocitosis, y los relacionados con la obtención de la muestra, normalmente la hemólisis y la coagulación [6].

Con respecto a la fase analítica, la primera fuente de error es la calibración del método. La siguiente es la interacción entre la muestra y el reactivo, ya sea debido a interferencias relacionadas con el paciente, a influencias indeseadas relacionadas con la muestra, o la existencia de un posible efecto matriz. Las siguientes fuentes potenciales de error en esta fase son la generación, interpretación y validación de los resultados [4]. No obstante, la mayoría de los autores coinciden en que los errores analíticos se pueden eliminar con el uso de la tecnología. De este modo, se debe centrar la atención en la totalidad del proceso POCT [7, 8].

En la fase final del proceso POCT (postanalítico), se pueden producir errores en el formato del informe, el informe de valores críticos o de otros resultados, así como en el último paso, la gestión de informes, que implica la verificación, preservación, almacenamiento y recuperación de informes [4].

Realizar un seguimiento de los indicadores de calidad en todas estas fases resulta muy útil a la hora de reducir errores en las POCT [8, 9].

El Instituto de Medicina de los EE.UU. establece dos tipos de errores médicos asociados a las POCT que pueden afectar a la seguridad del paciente: iniciar una actuación médica incorrecta y/o desconocer la trascendencia del resultado de la prueba, lo que deriva en que no se realicen las actuaciones necesarias [10]. Esta es otra manera de clasificar los errores en las POCT.

Las gasometrías son una de las pruebas POCT más utilizadas en los servicios de urgencias o en pacientes hospitalizados. La mayoría de las plataformas también miden otros parámetros como el potasio, la glucosa y el lactato, entre otros. Para estas plataformas se suele utilizar una muestra de sangre entera. Sin embargo, no debemos olvidar el riesgo de hemólisis, lo que suele ocurrir durante la extracción de la sangre, especialmente en escenarios clínicos con un elevado nivel de presión, como los servicios de urgencias o las UCI [11]. La hemólisis *in vitro* suele producirse durante la fase preanalítica, normalmente en el mismo proceso de extracción o durante el transporte de la muestra al laboratorio. Otros factores pueden también provocar hemólisis. Por ejemplo, algunos autores señalan un mayor riesgo de hemólisis en la sangre extraída a través de un catéter, frente a la obtenida mediante agujas de mariposa o agujas rectas [12].

También hay que tener en cuenta que, a diferencia de los analizadores químicos de los laboratorios centrales, que pueden cuantificar la concentración de hemoglobina libre en muestras de plasma y suero y proporcionan un índice de hemólisis, los analizadores de gases en sangre no pueden detectar la hemólisis en las muestras y, debido a las características de las mismas, esta tampoco se puede detectar a simple vista. Se han utilizado otras estrategias mediante algoritmos de inteligencia artificial, que pueden detectar errores analíticos mediante el reconocimiento de patrones en perfiles analíticos comunes, habiendo propuesto algunos autores un modelo analítico predictivo para marcar resultados elevados de potasio sospechosos [13]. La hemólisis puede alterar el resultado de parámetros como el potasio, dando resultados anormalmente elevados, o informando concentraciones normales que enmascaran niveles que, en realidad, son bajos (hipopotasemia). De este modo, reconocer la hemólisis cuando se utilizan métodos de POCT puede entrañar una gran dificultad.

## Hiperpotasemia y hemólisis

La hiperpotasemia se suele definir como una concentración de potasio en suero >5.5 mmol/L o en plasma >5.0 mmol/L [14]. La hiperpotasemia severa, definida habitualmente como niveles de potasio en suero o plasma >6.5–7.0 mmol/L se asocia a un mayor riesgo de arritmia cardíaca potencialmente letal y requiere actuación médica urgente [14]. La prevalencia de esta alteración analítica alcanza el 10% en los pacientes hospitalizados [6]. La hiperpotasemia suele ser asintomática y se suele detectar en las analíticas rutinarias. Cuando aparecen síntomas, estos son inespecíficos. Las alteraciones en el potasio plasmático pueden afectar a la función nerviosa, muscular y cardíaca, pudiendo provocar complicaciones graves como fasciculación, parestesia y arritmias (la hiperpotasemia grave puede provocar parálisis general). La hiperpotasemia puede producir alteraciones electrocardiográficas características, como ondas T en forma de pico o aplanadas, ausencia de ondas P, complejos QRS anchos, y ondas sinusoidales [15].

Cuando se evalúa a un paciente con hiperpotasemia, se debe revisar la historia médica para identificar posibles factores asociados. Se deberán comprobar otras posibles causas, como el fallo renal, la diabetes, la insuficiencia suprarrenal y el consumo de fármacos que provocan hiperpotasemia. Los análisis de sangre y orina deberán ir orientados a determinar parámetros que sean útiles a la hora de distinguir entre las causas renales y no renales de hiperpotasemia [16].

Sus causas se pueden dividir entre biológicas, como el fallo renal o un consumo o administración de niveles altos de potasio asociado a ciertos fármacos, como los beta bloqueadores, la digoxina y los diuréticos ahorradores de potasio. Otras causas de hiperpotasemia pueden estar relacionadas con problemas preanalíticos, como la hemólisis de la muestra, las presencia prolongada de un torniquete o haber apretado los puños repetidamente, la contaminación de la muestra a través de las vías de infusión, el almacenamiento prolongado de sangre centrifugada, así como la leucocitosis o la trombocitosis [14, 17, 18].

La hemólisis se define como la ruptura de los eritrocitos, que resulta en la liberación de sus componentes intracelulares en el plasma o el suero. La hemólisis se puede producir *in vitro* o *in vivo*, produciéndose la hemólisis en más del 2% de las muestras *in vitro* [19]. Podemos definir la hemólisis *in vitro* como la ruptura de los eritrocitos durante la extracción, gestión, transporte y almacenamiento de muestras biológicas [20]. Se trata de un problema notable en las pruebas diagnósticas, ya que los resultados obtenidos de muestras hemolizadas no son fiables.

El 98% del potasio que se encuentra en el organismo se halla en los fluidos intracelulares (a una concentración aproximada de 140 mmol/L), mientras que solo el 2% se encuentra en fluidos extracelulares (3.8–5.0 mmol/L) [21]. Esto explica por qué un pequeño porcentaje de la hemólisis puede tener un gran impacto en las concentraciones de potasio sérico o plasmático. La hemólisis es la causa más frecuente de pseudohiperpotasemia (hiperpotasemia espuria) o pseudonormopotasemia, pudiendo enmascarar los niveles bajos de una hipopotasemia y derivar en un diagnóstico erróneo y la consiguiente toma de decisiones médicas inadecuadas [22]. Algunos autores han alertado de que entre el 4% y el 13% de las muestras de sangre entera enviadas al laboratorio para gasometría presentan hemólisis oculta [21, 22]. En la Tabla 1 se enumeran causas frecuentes de pseudohiperpotasemia (hiperpotasemia espuria) o pseudonormopotasemia (normopotasemia espuria). Desde un punto de vista conceptual, estas causas se pueden clasificar en cuatro grupos: 1) causas relacionadas con la fase preanalítica de las POCT, especialmente aquellas asociadas a la extracción y manipulación de las muestras; 2) la fase analítica; 3) patologías relacionadas con el propio paciente; y 4) causas relacionadas con aspectos organizativos [25]. El mayor porcentaje de errores se produce en la fase preanalítica, especialmente durante la extracción de la muestra [23].

**Tabla 1:** Posibles causas de pseudohiperpotasemia o pseudonormopotasemia en las pruebas POCT.

Relacionadas con el proceso preanalítico – Un torniquete excesivo – Apretar los puños repetidamente – Tamaño inadecuado de la aguja (calibre estrecho) – Uso de una jeringa y aguja en lugar de un sistema de extracción al vacío – Lugar inusual de venopunción (esto es, otro que no sea la fosa antecubital) – Excesivo flujo sanguíneo (vacío) – Contaminación del tubo EDTA (por contaminación de la aguja u orden incorrecto de extracción) – Venopunción traumática (presión sobre la zona de punción) – Extraer la sangre de una vena en la que se ha infundido potasio – Mezcla de la muestra excesivamente vigorosa – Almacenamiento prolongado de sangre no centrifugada – Demora en el análisis (glicólisis *in vitro* en proceso, fuga de potasio) – Jeringa de gasometría transportada en contacto directo con hielo Relacionadas con el proceso analítico – Error analítico – Excesiva presión de la inyección al introducir la muestra en el analizador Relacionadas con el paciente – Leucocitosis severa (>150 × 10^9^/L) – Aumento notable del recuento de plaquetas (trombocitosis) (>500 × 10^9^/L) – Defectos heredados en la estructura de la membrana de los eritrocitos (glóbulos rojos quebradizos): antecedentes familiares de pseudohiperpotasemia y estomatocitosis hereditaria. – Pacientes de edad avanzada o recién nacidos Relacionadas con aspectos organizativos – Excesiva carga de trabajo y entorno bajo presión – Operadores insuficientemente capacitados

En los pacientes sin predisposición a la hiperpotasemia, puede que sea necesario medir los niveles de potasio en suero para descartar hiperpotasemia espuria, a menos que existan alteraciones electrocardiográficas que sugieran la necesidad de tratamiento urgente. No obstante, debemos ser conscientes de que las alteraciones electrocardiográficas no suelen estar relacionadas con el nivel de alteración en los niveles de potasio [14].

La prevalencia de muestras no adecuadas enviadas a gasometría oscila entre el 1.2% y el 3.7% [23]. Se ha demostrado que la hemólisis espuria afecta a varios parámetros. Algunos de ellos están descritos claramente en la literatura, como el aumento sustancial en las concentraciones de potasio debido al diferente gradiente de concentración entre las células sanguíneas y el plasma, o el decremento observado en la concentración de calcio ionizado, aunque otros parámetros, como pO_2_, pCO_2_, sO_2_ y COHb, también pueden verse afectados. Estas variaciones en los gases en sangre podrían deberse a complejos mecanismos patofisiológicos sin identificar, relacionados con la presencia de hemoglobina libre de células, así como con la liberación de una variedad de moléculas intracelulares [20]. Sería deseable que los fabricantes de analizadores de gases en sangre pudieran desarrollar nuevos dispositivos capaces de identificar la presencia de sustancias que interfieren en las muestras de sangre entera, como la hemólisis, la lipemia y la ictericia en química clínica y en las pruebas de coagulación en muestras séricas o plasmáticas.

Wilson y cols. publicaron en 2018 un estudio ambispectivo donde se compararon las tasas de hemólisis en muestras de gases en sangre obtenidas en unidades de cuidados intensivos frente a las obtenidas en los servicios de urgencias. El estudio demostró que la hemólisis era más frecuente y más sustancial en las muestras obtenidas en los servicios de urgencias que en las de la UCI, y superior en las muestras de sangre entera que en las muestras de suero extraídas tanto en el servicio de urgencias como en la UCI. El 13% de los valores de potasio determinados en el servicio de urgencias y el 4% de los determinados en la UCI con POCT en muestras de sangre entera se habrían considerado potencialmente inexactos debido a la hemólisis (leve o severa) [24].

## POCT vs. pruebas en el laboratorio central

Las anomalías electrolíticas pueden causar eventos fatales en urgencias. En este escenario, es esencial que se realice un análisis rápido de las alteraciones electrolíticas para garantizar una actuación médica inmediata. Las POCT reducen considerablemente el tiempo de respuesta cuando se emplean para medir los electrolitos en urgencias y en la UCI de adultos, frente a los análisis realizados en el laboratorio central [26]. Sin embargo, se han obtenido resultados contradictorios a la hora de analizar los valores exactos de los principales electrolitos, ya sea mediante POCT o con los métodos analíticos del laboratorio central [27], [28], [29]. Chako y cols. compararon el electrolito en sangre entera analizado con dispositivos POCT frente al electrolito en suero analizado en el laboratorio central. Los autores observaron diferencias notables en los valores, especialmente cuando los niveles de potasio eran <3 mmol/L. Sin embargo, se observó una diferencia uniforme y consistente en los valores de potasio >3 mmol/L [27]. Estas diferencias también pueden deberse a las características de los electrodos empleados para el análisis. Aunque la mayoría de los analizadores de POCT emplean electrodos para iones específicos (miden la actividad de los iones en plasma), los analizadores de laboratorio central están equipados con electrodos selectivos indirectos, que miden la actividad de los iones en una muestra prediluida, que puede verse afectada por la presencia de sólidos disueltos, como los triglicéridos o las proteínas. Morimatsu y cols. obtuvieron resultados similares de sodio y cloruro al comparar las diferencias en la brecha aniónica calculada con un analizador POCT de gases y electrolitos en sangre y con un analizador automático de química sanguínea de laboratorio central. Los autores concluyeron que esto podría provocar interpretaciones diferentes del estado ácido base y de los electrolitos [28]. Tal como se ha mencionado anteriormente, la clara desventaja de las POCT frente al laboratorio central es la limitada capacidad que tienen los dispositivos POCT a la hora de detectar la hemólisis, frente a los analizadores químicos convencionales.

## ¿Qué se puede hacer?

Si las comparamos con las pruebas de laboratorio central, las POCT pueden eliminar ineficiencias, aunque también se pueden producir errores preanalíticos, analíticos y postanalíticos derivados de errores humanos en la realización de las pruebas. Prevenir dichos errores y moderar su impacto en la seguridad del paciente contribuirá a mejorar los resultados clínicos y a reducir costes. Los programas POCT adecuadamente dirigidos y organizados por el laboratorio clínico pueden ayudar a lograrlo. También es necesario aplicar estrategias para identificar con precisión al operador que valide la muestra antes de realizar la prueba analítica, así como para garantizar un rendimiento adecuado mediante planes de garantía de calidad, y asegurar la transferencia de datos (conectividad) [6].

Para mejorar la calidad de las pruebas POCT, se recomienda el desarrollo, implementación y validación del rendimiento mediante indicadores de rendimiento relevantes y fiables. Su identificación y selección se basará en la visión, valores y objetivos de la organización. Las estrategias deberán ir encaminadas a abordar procesos en los que sea más factible prevenir errores que pudieran afectar a la seguridad del paciente, por lo que deberán ser seleccionadas basándonos en los riesgos a lo largo de todo el proceso analítico. Es perentorio realizar un esfuerzo multidisciplinar para alcanzar los objetivos de rendimiento deseados y mejorar los resultados clínicos. Mediante la evaluación de los indicadores de rendimiento se podría identificar un conjunto de indicadores de calidad y derivar en la implementación de acciones de mejora de las pruebas POCT dirigidas por el laboratorio clínico para lograr una mejor seguridad y asistencia al paciente [30, 31].

Resulta esencial que el personal encargado de obtener la muestra de sangre posea una formación adecuada y conozca los factores que pueden provocar hemólisis, así como es necesario implementar procedimientos de extracción estandarizados para minimizar las tasas de hemólisis y mejorar la fiabilidad de los resultados analíticos. También sería deseable mejorar la tecnología de los analizadores de gases en sangre. Los analizadores de gases en sangre avanzados normalmente incorporan cooxímetros que miden la hemoglobina total en sangre entera. Quizá, los avances en los algoritmos y métodos empleados para tal propósito permitirán desarrollar nuevos instrumentos capaces de identificar la presencia de sustancias que interfieren en las muestras de sangre entera como la hemólisis.

La adecuada formación de los profesionales sanitarios que realizan estas pruebas, combinada con el desarrollo de nuevos analizadores capaces de detectar la hemólisis, en el contexto de un programa de POCT adecuadamente organizado dirigido y organizado por el laboratorio clínico, son las tres claves para lograr detectar y reducir la hemólisis. La capacidad para minimizar su incidencia durante la fase preanalítica de POCT ayudaría a mitigar notablemente los efectos negativos en el manejo de los pacientes debido a la pseudohiperpotasemia o la pseudonormopotasemia derivadas de falsos niveles de potasio en las pruebas POCT.
